# A novel model of nephrotic syndrome results from a point mutation in *Lama5* and is modified by genetic background

**DOI:** 10.1016/j.kint.2021.10.031

**Published:** 2022-03

**Authors:** Sara Falcone, Thomas Nicol, Andrew Blease, Michael J. Randles, Elizabeth Angus, Anton Page, Frederick W.K. Tam, Charles D. Pusey, Rachel Lennon, Paul K. Potter

**Affiliations:** 1 Mammalian Genetics Unit, Medical Research Council Harwell Institute, Harwell Campus, Oxfordshire, UK; 2 Centre for Cellular and Molecular Physiology, University of Oxford, Oxford, UK; 3 British Heart Foundation, Centre of Research Excellence, Division of Cardiovascular Medicine, Radcliffe Department of Medicine, John Radcliffe Hospital, University of Oxford, Oxford, UK; 4 Wellcome Centre for Cell-Matrix Research, Division of Cell-Matrix Biology and Regenerative Medicine, School of Biological Sciences, Faculty of Biology Medicine and Health, The University of Manchester, Manchester Academic Health Science Centre, Manchester, UK; 5 Biomedical Imaging Unit, Faculty of Medicine, University of Southampton, Southampton, UK; 6 Centre for Inflammatory Disease, Department of Immunology and Inflammation, Imperial College London, London, UK; 7 Department Biological and Medical Sciences, Faculty of Health and Life Sciences, Oxford Brookes University, UK

**Keywords:** albuminuria, glomerulus, nephrotic syndrome, proteinuria, proteomic analysis

## Abstract

Nephrotic syndrome is characterized by severe proteinuria, hypoalbuminaemia, edema and hyperlipidaemia. Genetic studies of nephrotic syndrome have led to the identification of proteins playing a crucial role in slit diaphragm signaling, regulation of actin cytoskeleton dynamics and cell-matrix interactions. The laminin α5 chain is essential for embryonic development and, in association with laminin β2 and laminin γ1, is a major component of the glomerular basement membrane, a critical component of the glomerular filtration barrier. Mutations in LAMA5 were recently identified in children with nephrotic syndrome. Here, we have identified a novel missense mutation (E884G) in the uncharacterized L4a domain of LAMA5 where homozygous mice develop nephrotic syndrome with severe proteinuria with histological and ultrastructural changes in the glomerulus mimicking the progression seen in most patients. The levels of LAMA5 are reduced *in vivo* and the assembly of the laminin 521 heterotrimer significantly reduced *in vitro*. Proteomic analysis of the glomerular extracellular fraction revealed changes in the matrix composition. Importantly, the genetic background of the mice had a significant effect on aspects of disease progression from proteinuria to changes in podocyte morphology. Thus, our novel model will provide insights into pathologic mechanisms of nephrotic syndrome and pathways that influence the response to a dysfunctional glomerular basement membrane that may be important in a range of kidney diseases.

## Graphical abstract

**Figure:**
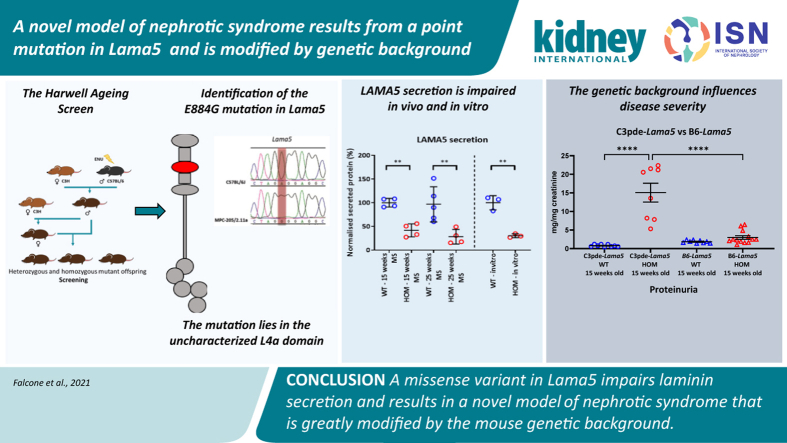

**Translational Statement**

The recent identification of mutations in *LAMA5* in pediatric patients affected by nephrotic syndrome indicates that this gene is important in human health and should be screened as a candidate in cases of nephrotic patients with no other diagnosis. The mutation identified in this study is the first murine model of a point mutation in laminin α5, demonstrating long-term proteinuria before kidney impairment, mimicking the progression seen in most patients. This model could serve as a unique tool to dissect disease mechanisms and test new treatments to alleviate symptoms.

Nephrotic syndrome (NS) is a clinical presentation characterized by severe proteinuria, reflecting dysfunction of the normally highly permselective glomerular filtration barrier. The other key phenotypes observed in NS include hypoalbuminemia, edema, hyperlipidemia, and lipiduria.^1^ Genetic studies of hereditary forms of NS have led to the identification of many genes encoding proteins, playing a crucial role in slit-diaphragm signaling, regulating actin cytoskeletal dynamics and cell-matrix interactions.^2^, ^3^, ^4^, ^5^ Most causative mutations occur in 1 of these 4 genes, *NPHS1*, *NPHS2*, *LAMB2*, and *WT1.*^6^ However, the causative allele remains unknown in 20% to 40% of cases.^7^^,^^8^

The glomerular basement membrane (GBM) is an unusually thick specialized extracellular matrix synthesized by both podocytes and endothelial cells.^9^ Laminins, major constituents of the GBM, are a family of self-assembling glycoproteins made up of at least 15 different αβγ heterometric macromolecules.^10^ The laminin heterotrimer exclusively present in the healthy mature GBM is laminin α5β2γ1 (laminin-521).^11^ Mutations in the *LAMB2* gene, encoding the laminin β2 chain, cause disorders with a wide clinical spectrum. Truncating mutations lead to Pierson syndrome, characterised by microcoria, congenital NS, muscular hypotonia, and neurodevelopmental defects,^12^, ^13^, ^14^ whereas missense variants cause a much milder variant of Pierson syndrome or isolated congenital NS.^15^^,^^16^

The laminin α5 chain, encoded by the *LAMA5* gene, is the only component of the GBM known to be essential for normal embryonic development, as shown by the lethality of the murine *Lama5* knockout.^17^ Laminin α5 consists of a short arm, starting with an N-terminal globular LN domain, followed by repeated rod-like regions consisting of multiple epidermal growth factor–like domains (LEa, LEb, and LEc domains) in combination with 2 additional globular domains (L4a and L4b domains).^11^ The long arm starts with a long coiled-coil domain that joins the α5 chain to the β and γ chains and ends with LG domain, which includes 5 globular modules.^11^^,^^18^ Interacting with integrin receptors, particularly integrin α3β1, laminin α5 facilitates anchorage and crosstalk between podocytes and the GBM.^18^, ^19^, ^20^, ^21^

A G3685R mutation in *LAMA5* has been reported in 2 independent studies of NS, although its pathogenicity has not been proven.^22^^,^^23^ More recently, 3 different homozygous genetic variants were identified in 3 families exhibiting early-onset NS between 18 months and 4 years of age, R747W, E1001G, and G2948.^24^

In other models of GBM dysfunction, disease progression is affected significantly by genetic background.^25^, ^26^, ^27^, ^28^ Although most backgrounds show a rapid disease progression, C57BL/6J slows disease. Herein, we show that the disease progression is slowed significantly in *Lama5*^E884G/E884G^ mice by genetic background, affecting early stages of disease and delaying proteinuria.

Herein, we describe a murine model of NS resulting from a mutation in *Lama5* affecting protein secretion both *in vivo* and *in vitro*. Our data add to the evidence for a potential pathogenic role of laminin α5 in the development of NS and emphasize the importance of modifiers in the progression of disease associated with GBM dysfunction.

## Methods

### Mice

C57BL/6J and C3H-C3pde6b^+^ inbred mice were maintained in the Mary Lyon Centre in Harwell, UK, in specific pathogen-free conditions. All animal procedures were performed under the guidance issued by the Medical Research Council in “Responsibility in the Use of Animals for Medical Research” (July 1993) and in accordance with Home Office regulations (Home Office Project Licence No. 30/3070).

The MUTA-PED-C3pde-205 mouse line was derived from a G_3_ pedigree produced in the Medical Research Council (MRC) Harwell N-ethyl-N-nitrosourea (ENU) mutagenesis screen, as described previously.^29^

### Mapping and next-generation sequencing

DNA from affected mice and littermate controls was tested on the Illumina Golden Gate “Mouse MD Linkage Panel” (Oxford Genomics Centre, Wellcome Trust Centre for Human Genetics). DNA from the G1 founder of the pedigree was sent for whole-genome sequencing employing the Illumina HiSeq platform (Oxford Genomics Centre, Wellcome Trust Centre for Human Genetics) and analyzed as previously described.^29^ The *Lama5* mutation was validated using Sanger sequencing (Source Bioscience).

### Clinical biochemistry analysis of plasma and urine

Blood samples were obtained through retro-orbital sinus with a prior i.p. injection of pentobarbital (Euthatal). Plasma concentrations of albumin, urea, creatinine, total cholesterol, high-density lipoprotein, and low-density lipoprotein were measured on an AU400 Olympus analyzer by the clinical chemistry core team at MRC Harwell.

Mice were singly housed overnight in metabolic cages (Techniplast) to collect urine for further analysis. Urine creatinine was quantified using an AU400 Olympus analyzer. Urinary protein concentration was quantified using Bradford protein assay (Biorad)^30^^,^^31^ and then normalized to urine creatinine.

Enzymatic method was used to measure creatinine levels in both serum and urine.

### Light and electron microscopy

For light microscopy, kidneys fixed in 10% neutral-buffered formalin were embedded in paraffin wax and sectioned at 5 μm. Kidney sections were stained with hematoxylin and eosin, periodic acid–Schiff, and Masson trichrome stain.

For transmission electron microscopy (TEM) and scanning electron microscopy (SEM), 1-mm^3^ cubes of kidney cortex were fixed in 3% glutaraldehyde and 4% formaldehyde in 0.1 M PIPES (the common name for piperazine-N,N′-bis(2-ethanesulfonic acid) buffer, pH 7.2 (minimum, 1 hour).

For TEM, specimens were then rinsed in 0.1 M PIPES buffer, postfixed in 1% buffered osmium tetroxide, rinsed in buffer, block stained in 2% aqueous uranyl acetate, dehydrated in an ethanol series, and embedded in TAAB resin (TAAB Laboratories). Gold–silver sections were cut, stained with Reynolds lead stain, and viewed on a Hitachi HT7700 transmission electron microscope.

For SEM, samples were then dehydrated through increasing strength of ethanol solutions and critical point dried using an Emitech K850 (EM Technologies LTD). Three specimens per animal were then mounted on stubs using silver paint (Agar Scientific) and sputter coated with platinum using a Quorum Q150T sputter coater (Quorum Technologies). The specimens were untimely visualized with a JEOL LSM-6010 (Jeol Ltd.).

### Cloning and expression in mammalian cells of the full-length laminin α5 chain

*Lama5* was cloned into a pCMV6-AC-His vector. The E884G mutation was introduced using site-directed mutagenesis kit (NEB). HEK-293 cells stably expressing human *LAMB1* and *LAMC1*^32^ were plated in 6-well plates 16 to 24 hours before transfection of pCMV6-AC-His-*Lama5* and pCMV6-AC-His-*Lama5*^E884G^ using jetPRIME (Polyplus). After 72 hours, conditioned medium was harvested, and cells were lysed. Samples of medium and cell lysate were run on 3% to 8% Tris-Acetate gels (Invitrogen) and transferred to hybond polyvinylidene difluoride membrane (GE Healthcare). After blocking with 5% w/v milk, membranes were incubated with anti-6XHis antibody (Origene; 1:1000) overnight. Goat–anti-rabbit IRDye680LT (1:15,000; LI-COR Biosciences) was used as secondary antibody, and fluorescent blots were scanned using a LI-COR Odyssey SA scanner (LI-COR Biosciences). The amount of secreted laminin α5 protein detected in the medium was normalized to the amount of laminin α5 protein detected in the corresponding cell lysate. Considering the average ratio of the controls to be equal to 100%, the secretion of the mutant samples was expressed as a percentage of the control sample secretion.

### Murine glomeruli isolation, protein extraction, and mass spectrometry analysis

The glomeruli from 15-week-old mice (wild type, n = 4; and homozygotes, n = 4) and 25-week-old mice (wild type, n = 5; and homozygotes, n = 4) were isolated following a modified Dynabeads-based protocol previously described.^33^ Enrichment of glomerular extracellular matrix was performed as previously described.^34^ The samples obtained by protein extractions were prepared for liquid chromatography–tandem mass spectrometry (MS) analysis: briefly, samples were resolved by sodium dodecylsulfate–polyacrylamide gel electrophoresis and visualised with Coomassie staining. The gel samples were cut into slices and then into 1-mm^3^ pieces and given to the MS facility core at the University of Manchester for in-gel proteolytic digestion, offline peptide desalting, and actual MS run. All the samples were run blindly, in a random sequence of genotype and age. Quantitative analysis was performed using the software using Progenesis LCMS (Non Linear Dynamics Ltd.) in association with the use of Mascot (Matrix Science) to identify the proteins.^35^ Statistical analysis was performed on proteins identified by at least 3 unique peptides. Protein interaction network analysis was performed using Cytoscape and the plug-in EnrichementMap.

## Results

### Identification of the *Lama5*^E884G^ mutation

**Figure 1:**
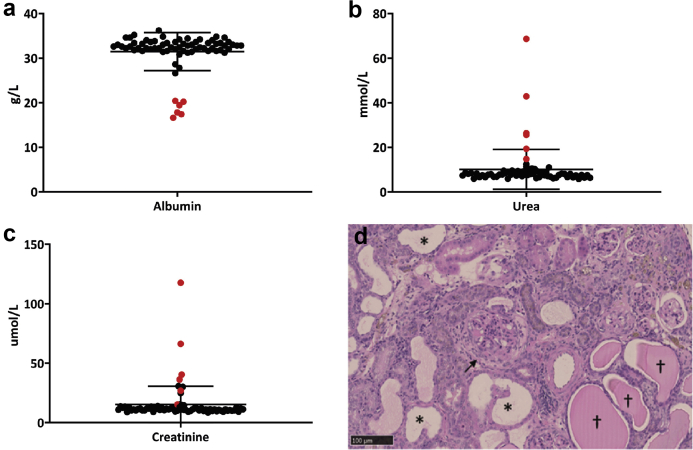
**(a–c) Clinical chemistry analysis of the pedigree MUTA-PED-C3pde-205 at 6 months of age.** Outliers (in red) exhibit lower levels of plasma albumin (**a**) and higher levels of urea (**b**) and creatinine (**c**) when compared with the other mice of the pedigree. (**d**) Hematoxylin and eosin–stained sections of kidneys of affected mice showed typical lesion of chronic kidney disease: fibrosis of the Bowman capsule (black arrow) and dilated tubules (stars) with protein casts (crosses). Bar = 100 μm. To optimize viewing of this image, please see the online version of this article at www.kidney-international.org.

As part of a phenotype-driven mutagenesis screen,^29^ we identified mice with plasma albumin levels 2 SDs below littermate range at 6 months of age (Figure 1a) and increased levels of urea and creatinine (Figure 1b and c). The health of affected mice gradually deteriorated, and they reached welfare limits around 248 ± 13.7 days of age (mean ± SEM). Postmortem histologic analysis confirmed the presence of chronic kidney disease (Figure 1d).

**Figure 2:**
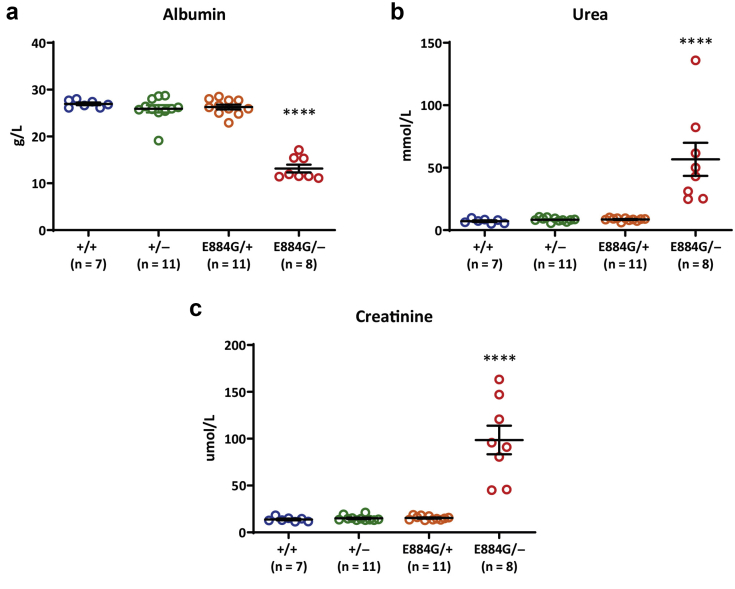
**The clinical chemistry analysis of plasma confirmed hypoalbuminemia (a) and renal dysfunction (b,c) only in *Lama5***^**E884G/–**^**compound heterozygote mice at 22 weeks of age.** The values shown are means ± SEM. One-way analysis of variance was used. ∗∗∗∗*P* < 0.0001.

Single-nucleotide polymorphism mapping localized the mutation to a region between 171 Mb and the distal end of chromosome 2 (Supplementary Figure S1A). Through whole-genome sequencing, a high confidence candidate mutation was identified in the gene *Lama5*, encoding laminin α5 (Supplementary Table S1). The ENU-induced point mutation (c.2651A>G) results in a glutamic acid to a glycine substitution at amino acid 884 (Supplementary Figure S1B). The glutamic acid residue, conserved back to Drosophila (Supplementary Figure S1C), is part of the L4a domain on the short arm of laminin α5^36^ and was predicted to be deleterious for protein function and/or structure (Supplementary Table S2). Retrospective genotyping confirmed only homozygous mutant mice exhibited evidence of kidney dysfunction, with no phenotype observed in heterozygous animals (Supplementary Figure S1D–F). To confirm the variant in *Lama5* was the causative mutation, *Lama5* knockout mice^37^ were crossed with *Lama5*^E884G/E884G^ mice to generate compound heterozygotes (*Lama5*^E884G/–^). Heterozygous *Lama5*^E844G/+^ mice and *Lama*5^+/–^ mice did not show any impairment in renal function at 22 weeks of age (Figure 2a–c). However, Lama5^E884G/–^ compound heterozygotes developed increased levels of plasma urea and creatinine, with significantly reduced plasma albumin levels (Figure 2a–c), thus confirming the *Lama5*^E884G^ ENU-induced mutation as the causative allele.

### Phenotype modification

**Figure 3:**
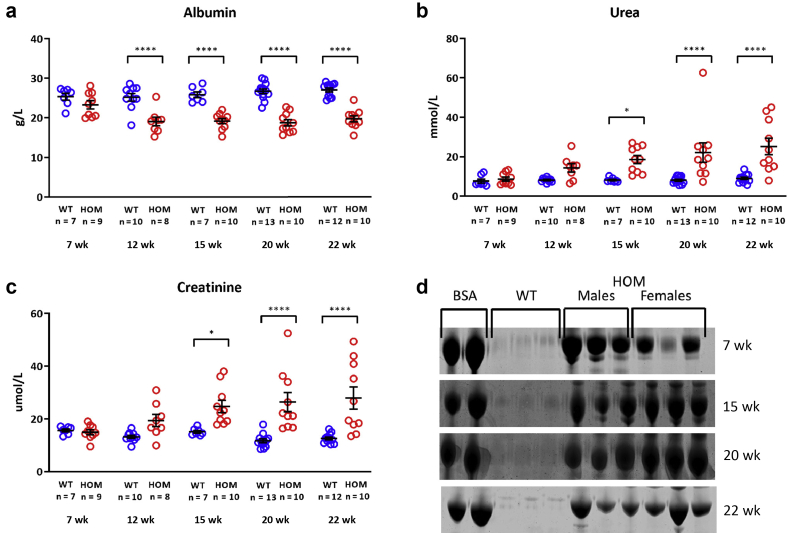
**Clinical chemistry analysis of plasma of mice backcrossed 1 generation to the C3H background (C3pde-B6-*Lama5* line) shows worsening of renal function, with low levels of albumin (a) and high urea (b) and creatinine (c) levels from 12 to 15 weeks of age.** Hypoalbuminemia is the earliest detectable change in the plasma at 12 weeks of age. (**d**) Coomassie blue–stained sodium dodecylsulfate–polyacrylamide gel electrophoresis gels of urine samples reveal the presence of proteinuria in males and females from 7 weeks of age. The values shown are means ± SEM. Two-way analysis of variance with Bonferroni *post hoc* test was used. ∗*P* < 0.05, ∗∗∗∗*P* < 0.0001. BSA, bovine serum albumin; HOM, homozygous; WT, wild type.

The mice identified as abnormal are on a mixed genetic background of C57BL/6J and C3H-C3pde6b^+^,^29^ and these were then bred with C3H-C3pde6b^+^ mice. A time course of mice backcrossed 1 generation to the C3H-C3pde6b^+^ background (C3pde-B6-*Lama5* line) revealed hypoalbuminemia as early as 12 weeks and increased plasma levels of creatinine and urea at 15 weeks (Figure 3a–c). Proteinuria was detected at 7 weeks of age (Figure 3d).

**Figure 4:**
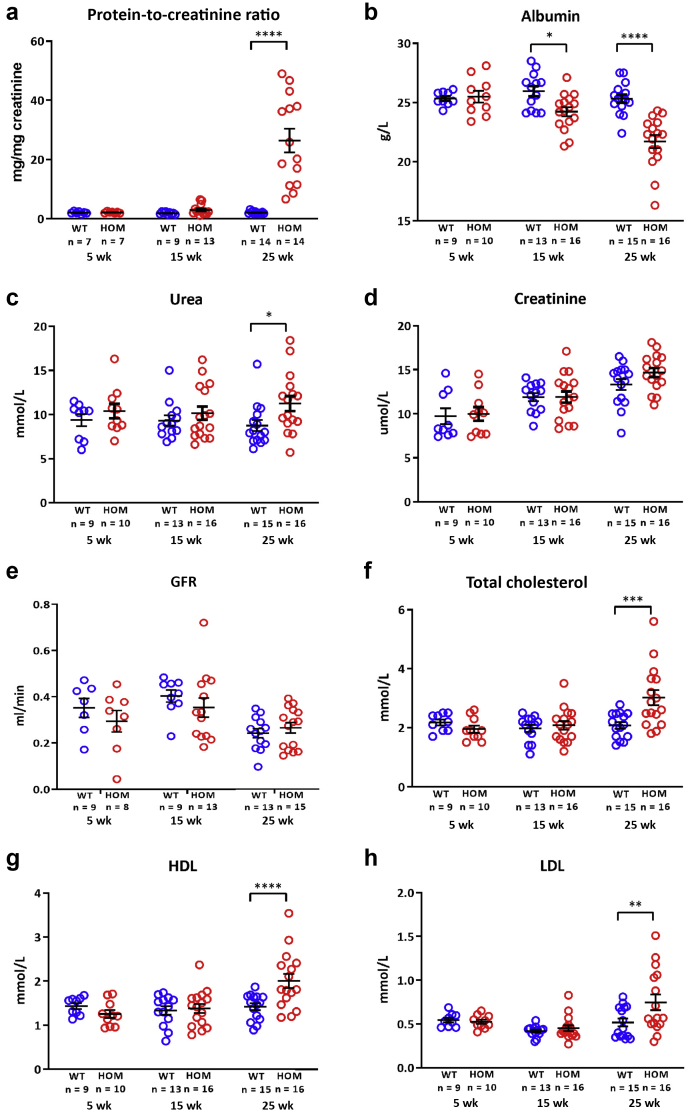
**Clinical chemistry analysis of urine and plasma of mice backcrossed 10 generations to the B6 background (B6-*Lama5***^**E884G/E884G**^**).** (**a**) Urinalysis showed proteinuria at 25 weeks. (**b**–**d**) There is significant hypoalbuminemia from 15 weeks (**b**), urea is slightly more elevated in homozygous (HOM) plasma at 25 weeks (**c**), but there is no difference in creatinine levels (**d**). (**e**) Creatinine clearance, an indicator of glomerular filtration rate (GFR), is normal in mutant mice at all ages tested. (**f**–**h**) Nonetheless, B6-*Lama5*^E884G/E884G^ mice exhibited a nephrotic phenotype with hypercholesterolemia in addition to hypoalbuminemia at 25 weeks. The values shown are means ± SEM. Two-way analysis of variance with Bonferroni *post hoc* test was used. ∗*P* < 0.05, ∗∗*P* < 0.01, ∗∗∗*P* < 0.001, and ∗∗∗∗*P* < 0.0001. HDL, high-density lipoprotein; LDL, low-density lipoprotein; WT, wild type.

We also bred the mutation onto the C57BL/6J background, and early backcross results suggested a delayed disease on the C57BL/6J background, as we and others have previously observed in models of GBM dysfunction.^25^^,^^26^^,^^34^^,^^38^ We therefore decided to make the *Lama5*^E884G^ mutation congenic on the C57BL/6J background to give us the best opportunity to study disease progression (B6-*Lama5*^E884G/E884G^ mice). This resulted in a delay of the observed phenotypes. The presence of protein in the urine was assessed using the urinary protein-to-urinary creatinine ratio method; results showed proteinuria at 25 weeks in the homozygotes (Figure 4a), whereas the backcross one C3pde-B6-*Lama5*^E884G/E884G^ mice exhibited significant proteinuria at 7 weeks (Figure 3d).

Kidney function seemed to be conserved, as indicated by plasma creatinine concentration (Figure 4d) and creatinine clearance rate (Figure 4e) in B6-*Lama5*^E884G/E884G^ mice up to 25 weeks of age, although a small but significant increase in urea was observed (Figure 4c).

**Figure 5:**
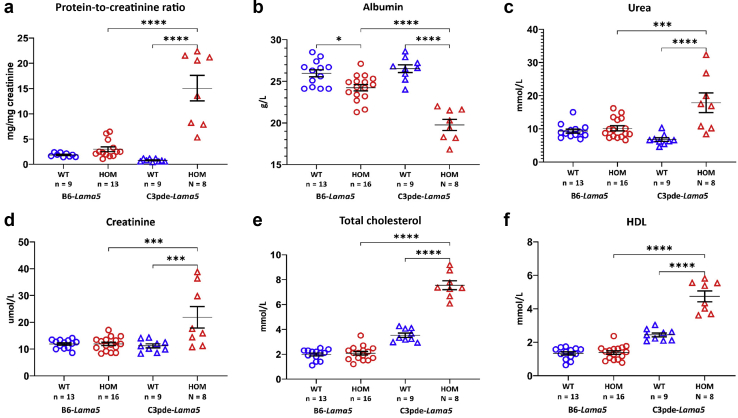
**A summary of the differences between the 2 congenic mouse stains.** Compared with B6-*Lama5*^E884G/E884G^, congenic C3pde-*Lama5*^E884G/E884G^ mice show proteinuria (**a**), increased hypoalbuminemia (**b**), and elevated urea (**c**), creatinine (**d**), total cholesterol (**e**), and high-density lipoprotein (HDL; **f**) starting from 15 weeks of age. HOM, homozygous; WT, wild type.

For a direct comparison between strains, cohorts of congenic *Lama5*^E884G^ C3H-C3pde6b^+^ mice (C3pde-*Lama5* ^E884G/E884G^) and *Lama5*^E884G^ C57BL/6J congenic mice (B6-*Lama5* ^E884G/E884G^) were bred and aged to 15 weeks. In C3pde-*Lama5*^E884G/E884G^ mice, hyperlipidemia, proteinuria, and increased levels of plasma urea and creatinine were observed at 15 weeks of age, but not in B6-*Lama5*^E884G/E884G^ mice (Figure 5). Homozygous mutant mice on both backgrounds exhibited hypoalbuminemia, but albumin levels were significantly lower in C3pde-*Lama5* ^E884G/E884G^ mice at this time point (Figure 5b). These results indicate B6-*Lama5*^E884G/E884G^ congenic mice exhibit a more slowly progressing NS, and that disease was delayed compared with the C3pde-*Lama5* ^E884G/E884G^ congenic mice. A summary of the differences between the 2 strains is shown in Supplementary Table S3.

**Figure 6:**
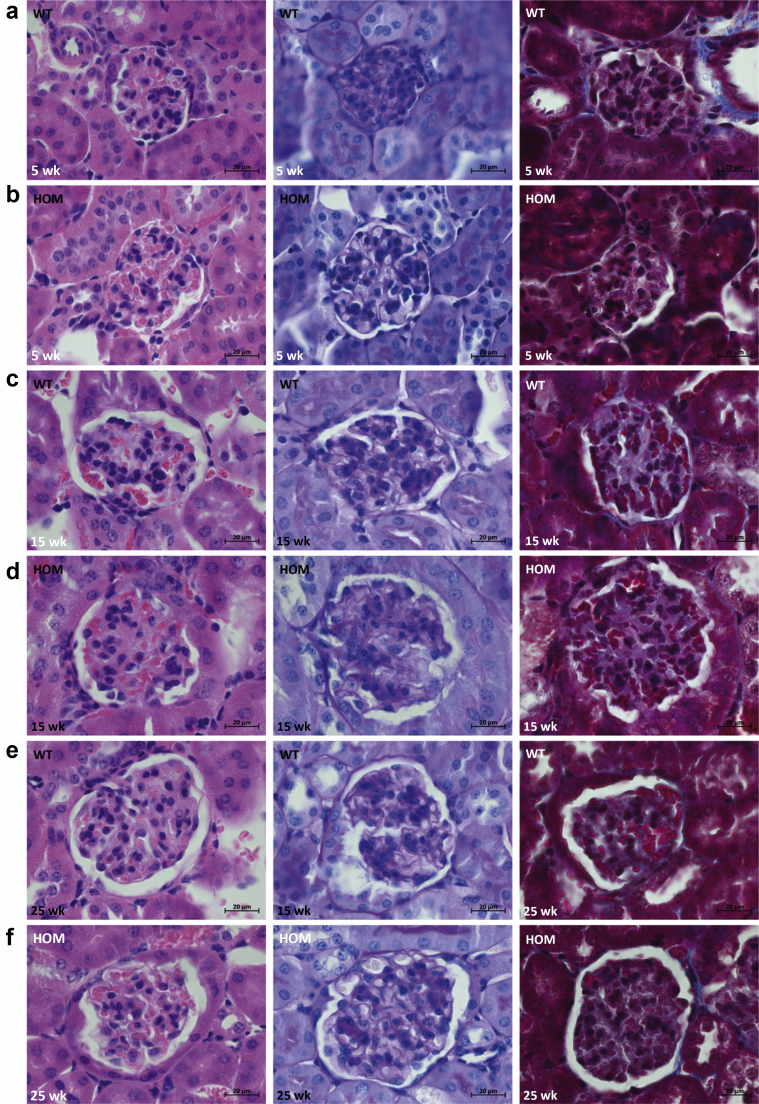
**Results of the histologic time course study.** Hematoxylin and eosin (first column), periodic acid–Schiff (middle column), and Masson trichrome (right column) stains were used to study kidney architecture in B6-*Lama5* mice. No overt differences were identified between wild-type (WT) controls (**a**, 5 weeks; **c**, 15 weeks; and **e**, 25 weeks) and affected mice (**b**, 5 weeks; **d**, 15 weeks; and **f**, 25 weeks). Bar = 20 μm. HOM, homozygous. To optimize viewing of this image, please see the online version of this article at www.kidney-international.org.

**Figure 7:**
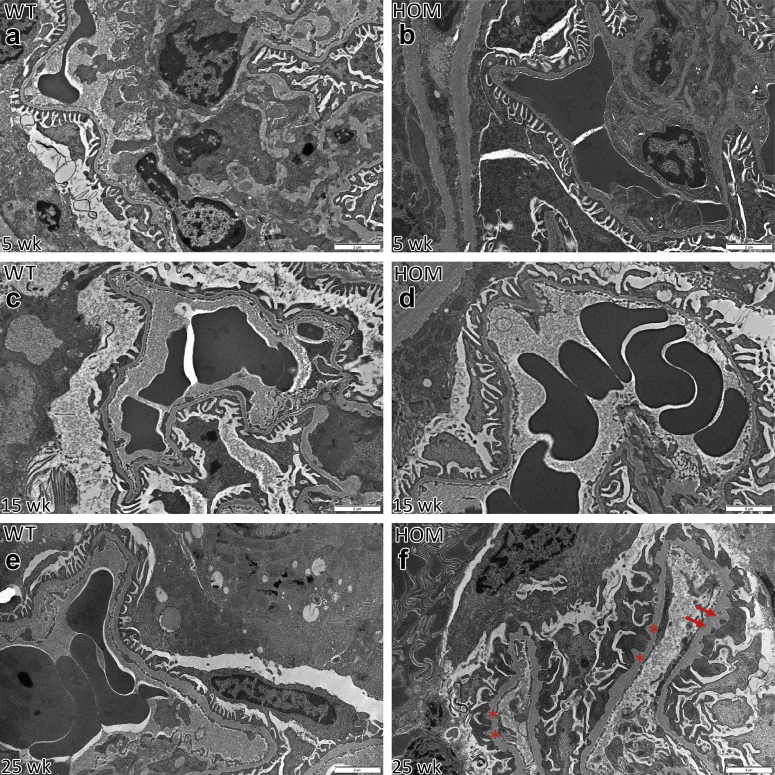
**Results of transmission electron microscopy time course study on wild-type (WT) and B6-*Lama5***^**E884G/E884G**^**kidneys.** The glomerular basement membrane (GBM) maintains its ribbon-like appearance, and podocyte foot processes are clearly distinguishable in both WT (**a,c**) and homozygous (HOM) mice at 5 and 15 weeks (**b,d**). At 25 weeks of age, mutant mice (**f**) developed irregular GBM (stars), loss of foot process definition, partial fusion (foot process effacement), and podocyte foot process invasion of the GBM (arrows), whereas aged matched WT mice maintain a normal appearance of the glomerular filtration barrier (**e**). Bar = 2 μm. To optimize viewing of this image, please see the online version of this article at www.kidney-international.org.

**Figure 8:**
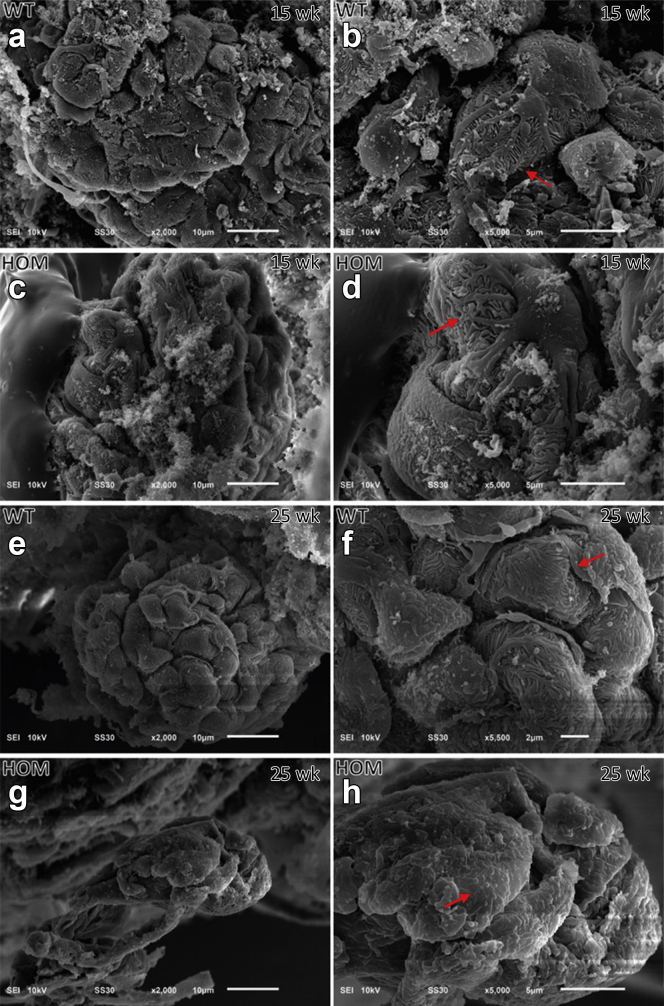
**Results of scanning electron microscopy time course study on wild-type (WT) and B6-*Lama5***^**E884G/E884G**^**kidneys.** A normal podocyte body, primary processes, and interdigitate foot processes can be observed in WT mice at 15 weeks (**a,b,** arrows) and 25 weeks (**e,f,** arrows), and in homozygous (HOM) mice at 15 weeks (**c,d,** arrows). Foot process effacement, with loss of any interdigital structure, is present in homozygotes at 25 weeks of age, confirming the transmission electron microscopy data (**g,h,** arrow). To optimize viewing of this image, please see the online version of this article at www.kidney-international.org.

Compared with unaffected controls, kidneys of B6-*Lama5* ^E884G/E884G^ mice did not show any glomerular (Figure 6) or tubular (data not shown) lesions by light microscopy. Normal renal ultrastructure was observed by TEM in homozygotes at 5 and 15 weeks, but irregular GBMs and podocyte foot process effacement were present at 25 weeks only in homozygous B6-*Lama5*^E884G/E884G^ mice (Figure 7). The GBM also exhibited signs of focal thickening, and podocyte invasion into the GBM was also evident, in keeping with previous studies of glomerular injury.^39^ Foot process effacement was confirmed by SEM (Figure 8). At 25 weeks, the foot processes from homozygotes appeared completely flattened. No difference was seen at 15 weeks with either TEM or SEM.

**Figure 9:**
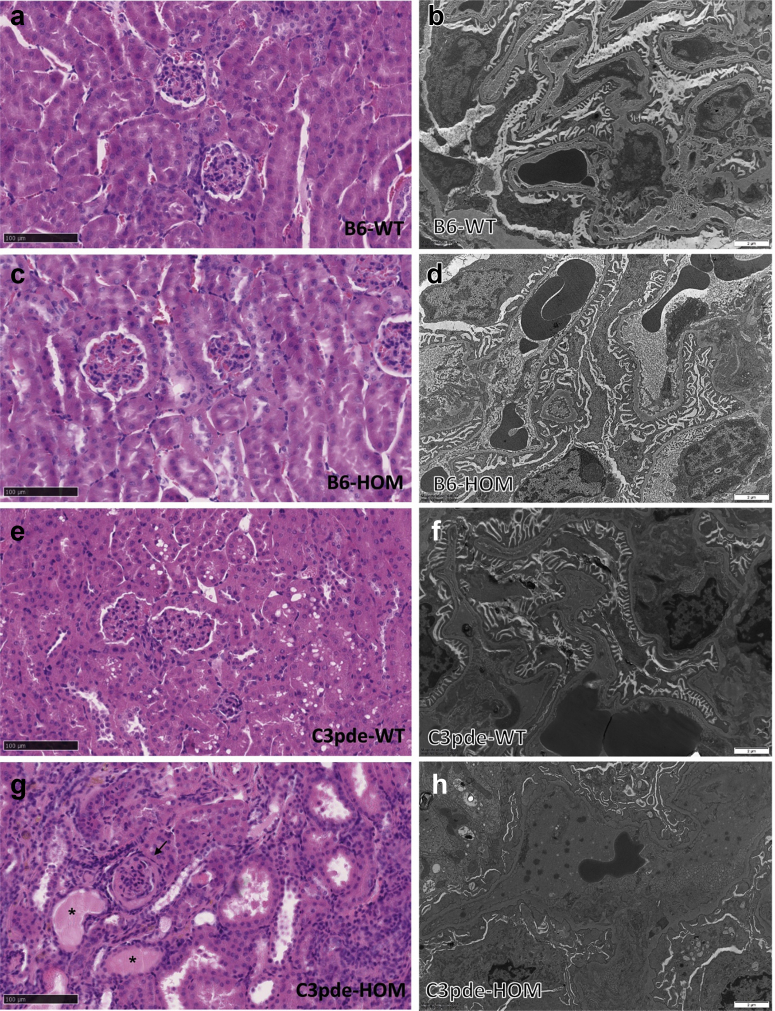
**(a–d) Histologic and transmission electron microscopy comparison between the 2 lines shows no differences between wild-type (WT) controls and homozygous (HOM) B6-*Lama5* mice.** (**e,f**) Similar results were obtained for WT C3pde littermate controls. (**g,h**) On the contrary, 15-week-old C3pde-*Lama5*^E884G/E884G^ mice displayed dilated tubules with protein casts (stars) and fibrotic glomeruli (arrow; **g**) and foot process effacement (**h**). To optimize viewing of this image, please see the online version of this article at www.kidney-international.org.

On the contrary, C3pde-*Lama5*^E884G/E884G^ mice displayed histologic features of chronic kidney disease and complete foot process effacement at 15 weeks of age (Figure 9).

### Impaired laminin α5 secretion *in vitro*

**Figure 10:**
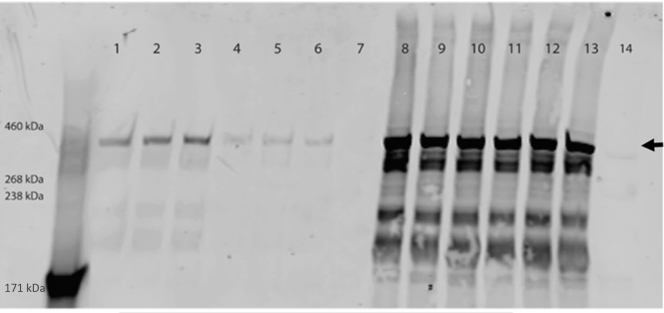
**Screening of secreted and cell expressed full-length 6xHis-tagged laminin α5 transfected in HEK293 cells stably expressing human laminin β1 and human laminin γ1.** Control pCMV6-LAMA5 construct (arrow; expected size, 404 kDa) could be detected as secreted protein in the medium (lanes 1, 2, and 3) and expressed in the cell lysate (lanes 8, 9, and 10). In presence of the E884G mutation, protein abundance in the medium was dramatically reduced (lanes 4, 5, and 6), even though LAMA5 is expressed in cell lysate (lanes 11, 12, and 13). Untransfected HEK293 cells (lane 14) and their medium (lane 7) were used as negative control.

Simultaneous overexpression of wild-type and E884G *Lama5* in HEK293 cells with stable expression of *Lamb1* and *Lamc1*^32^ allowed for the secretion of both wild-type and mutant *LAMA5*, confirming that the mutant protein is secreted from the cell. Nevertheless, in the presence of the E884G mutation, laminin α5 protein secretion was reduced by 69.9% ± 9% (mean ± SEM), suggesting that the mutation E884G affects protein folding and results in a reduced laminin-521 assembly (Figure 10).

### Altered glomerular matrisome

**Figure 11:**
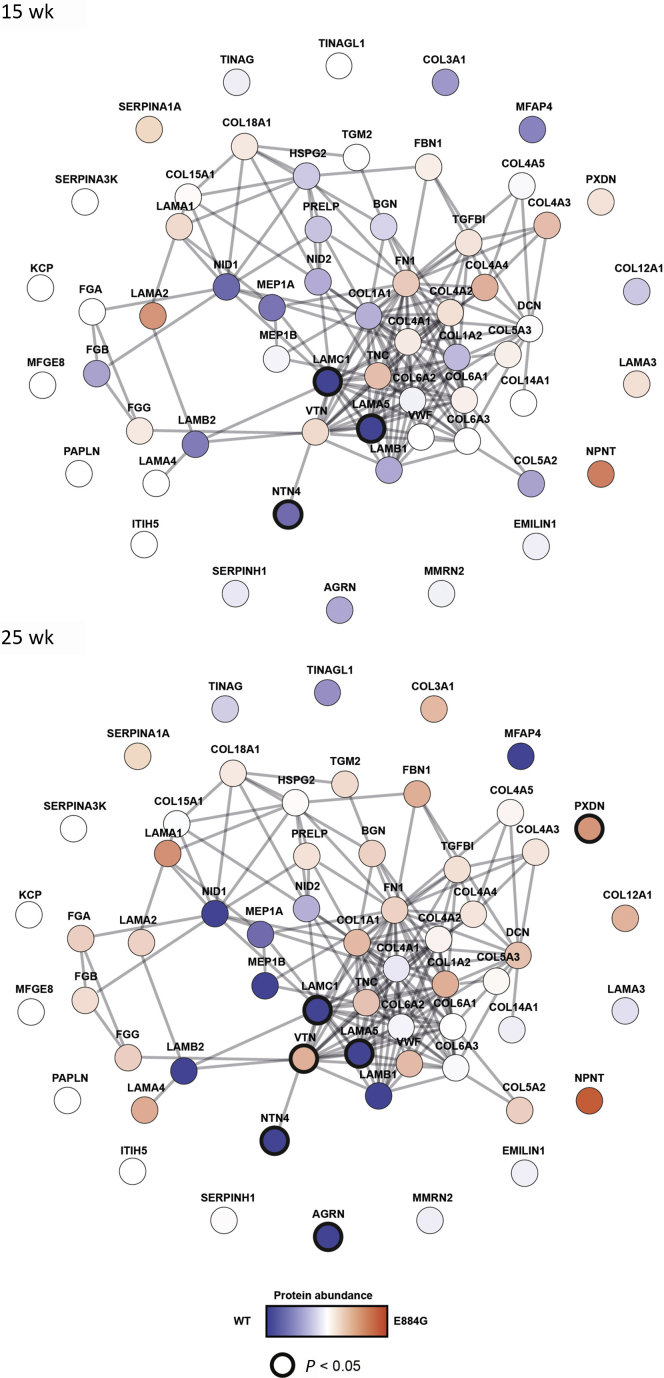
**Protein interaction network constructed from enriched glomerular extracellular matrix proteins identified by mass spectrometry.** The nodes, circles, represent the proteins identified; and the edges, lines, represent a reported protein–protein interaction. Nodes are colored according to the protein abundance, blue if enriched in the wild-type samples and red if enriched in the mutant samples. Darker circles around the nodes indicate statistical significance (2-way analysis of variance with Sidak *post hoc* test *P* < 0.05). LAMA5, LAMC1, and NTN4 show reduced abundance in homozygotes at 15 and 25 weeks. Agrin (AGRN) abundance is lower at 25 weeks, whereas vitronectin (VTN) is increased at 25 weeks. WT, wild type.

**Figure 12:**
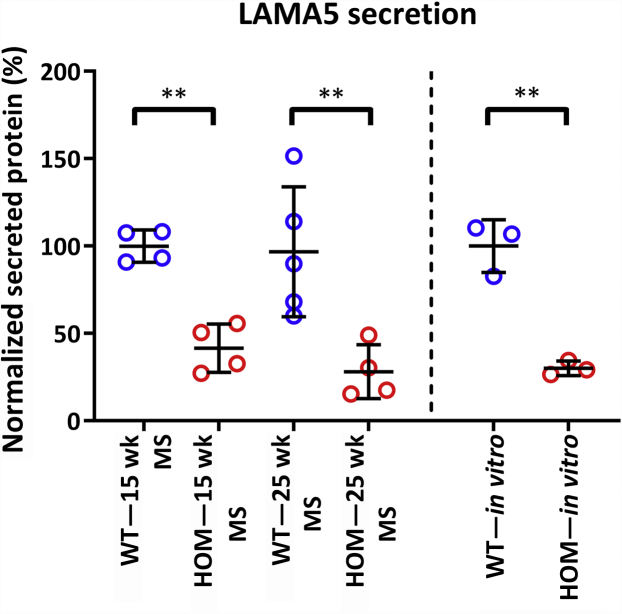
**Comparison of LAMA5 protein abundance in extracellular matrix (ECM) fraction and *in vitro* experiment expressed in percentage.** ECM fractions were normalized to 15 weeks wild type (WT) and *in vitro* experiment, as described in Figure 8. The values shown are means ± SEM. ECM fraction: 2-way analysis of variance with Bonferroni *post hoc* test: ∗∗*P* < 0.01. *In vitro* experiment: unpaired parametric *t*-test: ∗∗*P* < 0.01. HOM, homozygous; MS, mass spectrometry.

For further analyses, we focused on the B6-*Lama5*^E884G/E884G^ mice because the longer time course of disease presented the best opportunity to identify changes over time that may relate to the mechanisms underlying disease. Matrix composition has been shown to affect podocyte responses.^39^ To examine GBM composition *in vivo*, MS analysis was performed on the extracellular matrix fraction of glomeruli isolated from 15- and 25-week-old wild-type and B6-*Lama5*^E884G/E884G^ mice (Figure 11). The time points were chosen as they represent stages of disease that were asymptomatic and when symptoms were apparent. Statistical analysis on MS data showed a significant reduction of 58.4% ± 14.8% and 68.6% ± 14.1% (mean ± SEM) in the abundance of laminin α5 in B6-*Lama5*^E884G/E884G^ samples at 15 and 25 weeks, respectively, with no statistically significant changes between the 2 time points, supporting the *in vitro* results (Figure 12). Laminin γ1 showed similarly reduced abundance at both time points. Other proteins related to the laminin-521 trimer were less abundant in B6-*Lama5*^E884G/E884G^ glomeruli, such as agrin (25 weeks) and netrin-4 (15 and 25 weeks), whereas vitronectin abundance increased in mutants at 25 weeks. Netrin-4 is a protein of the laminin-related netrin family, with a structure related to laminin β chains, and expressed.^40^ It interacts with laminin γ1, forming a high-affinity complex. The strong bond between the LN domains of both proteins prevents the polymerization of the laminin trimers and disrupts the preexisting laminin network in a nonenzymatic manner.^41^

We found decreased expression of agrin, the most abundant heparan sulphate proteoglycan in the GBM,^42^ only at 25 weeks, coinciding with the development of symptoms. Although neither agrin nor the other heparan sulphate proteoglycans of the GBM are considered essential to maintaining normal barrier function,^43^ the absence or reduction of heparan sulphate proteoglycans triggers the local amplification of C3 activation, worsening or exacerbating glomerular lesions.^44^^,^^45^

Vitronectin was more abundant in homozygous mice at 25 weeks. Vitronectin is expressed in the glomerulus, and patients with glomerular diseases show increased accumulation in both glomeruli^46^ and urine^47^; however, it seems to only have a minimal impact on fibrogenesis.^48^ Taken together, these results suggest a reduction of the laminin network as a whole in the *Lama5*^E884G/E884G^ GBM, with a gradual reduction of the levels of some proteins, reflecting an increased severity of disease and possibly contributing to inflammatory processes.

## Discussion

Herein, we describe the initial findings in a novel model of NS with a mutation in the gene coding laminin α5 chain. Phenotypically, C3pde-*Lama5*^E884G/E884G^ mice showed typical signs of NS, such as severe proteinuria, hypoalbuminemia, and hypercholesterolemia.^1^ LAMA5 is the most abundant laminin α chain of the body, and it is expressed almost ubiquitously, hence the dramatic phenotype displayed by *Lama5* knockout mice.^17^ Even given this crucial role, the mutation E884G of laminin α5 ultimately leads only to NS, without the presence of other obvious phenotypes. Histologic and ultrastructural studies on the congenic B6-*Lama5*^E884G/E884G^ revealed a delayed disease progression with the absence of lesions by light microscopy up to 25 weeks. Although there was mild hypoalbuminemia at 15 weeks, by TEM and SEM, foot process effacement was visible at 25 weeks but not at 15 weeks, coinciding with detectable proteinuria.

How the GBM plays a role in the filtration of plasma is still unclear. A popular hypothesis is that the deposition of ectopic laminins, such as LAMA1, LAMA2, and LAMB1, leads to a change of the usual gel properties and porosity of the GBM.^49^ Accumulation of ectopic laminins occurs not only in the case of *LAMB2*-related disease, but also in Alport syndrome (another GBM disease caused by mutations in one of the collagen type IV chains), where deposition of the laminin α2 chain in GBM causes an increased phosphorylation of focal adhesion kinase (FAK).^50^ However, B6-*Lama5*^E884G/E884G^ samples did not show increased abundance or staining of ectopic laminins, suggesting that the filtration role of the GBM does not depend solely on the aberrant presence of constituent proteins.

We noted that disease progression was greatly affected by genetic background. Homozygous mice of the original mixed background pedigree exhibited signs of kidney impairment at 6 months and had to be sacrificed at around 8 months of age, presumably because of end-stage kidney disease. On the contrary, B6-*Lama5*^E884G/E884G^ mice only developed proteinuria at 25 weeks, but this was not associated with impaired kidney function (Supplementary Figure S2). A direct comparison of congenic C3pde-*Lama5*^E884G/E884G^ and B6-*Lama5*^E884G/E884G^ mice at 15 weeks of age showed an accelerated disease progression in the C3pde-*Lama5*^E884G/E884G^ mice, with proteinuria, hypoalbuminemia, hypercholesterolemia, and ultrastructural changes in the kidney, whereas the only detectable phenotype in B6-*Lama5*^E884G/E884G^ congenic mice was a mild hypoalbuminemia. In our *Lama5* lines, as in the LAMB2-Del44 mice,^16^ there is a high level of proteinuria, but the B6-*Lama5*^E884G/E884G^ mice show a delay in the development of proteinuria, suggesting the modifier(s) are influencing an early response to GBM dysfunction rather than the response to proteinuria.

Protein composition and organization of the GBM varies depending on the genetic background. A global proteomics analysis on the glomerular matrisome found that proteins such as netrin-4 and fibroblast growth factor 2 were enriched in FVB (an albino, inbred laboratory mouse strain that is named after its susceptibility to Friend leukemia virus B) glomeruli (a strain more susceptible to kidney disease^25^), whereas proteins such as tenascin C and type I collagen are enriched in C57BL/6J glomeruli.^34^^,^^35^ However, the susceptible/protective effect of the background mouse strain is consistent regardless of the glomerular filtration barrier component that is defective, as in the case of Alport syndrome,^26^^,^^38^ laminin-521,^51^ or genes associated with focal adhesion complexes, such as CD151.^25^

*In vitro* studies showed that the E884G mutation, in the uncharacterized LAMA5 L4a domain, results in a hypomorph that affects protein secretion to a similar level as determined in the proteomic assessment of the matrisome. Expression of the mutant laminin α5 chain in HEK293 cells expressing the laminin β1 and γ1 chains resulted in a reduced secretion of the LAMA5 heterotrimer.

Using proteomic analysis, we examined the glomerular GBM composition. MS analysis detected reduced abundance of laminin α5 and laminin γ1 in mutant samples at 15 and 25 weeks, possibly due to reduced secretion or reduced stability of the mutant protein. Decreased levels of laminin α5 were not observed with immunofluorescence, possibly due to insufficient sensitivity (Supplementary Figure S3). These data provide insight into 2 aspects of the mechanism for disease in *Lama5*^E884G/E884G^ animals. First, *Lama5*^E884G^ is capable of interacting with other laminin chains to some degree, as was observed in the LAMB2-Del44 mice,^16^ allowing trimer formation. Conversely, we observed that *Lama5*^E884G^ secretion is significantly reduced in comparison to wild types, both *in vitro* and *in vivo*.

Reduced secretion of the laminin-521 heterotrimer has also been observed in laminin β2-related NS, in particular due to mutations such as R246Q^52^ and C321R,^53^ and is associated with mild Pierson syndrome symptoms. Funk *et al.* also observed a reduction in the expression of the laminin-521 trimer, although to a greater degree than in our model.^16^ This may indicate that the level of reduction in laminin-521 heterotrimer correlates with the severity of disease, as our mutant results in a smaller reduction in laminin-521 and has a milder disease. Reduction of wild-type laminin α5 has previously been investigated by Shannon *et al.*, who described a more severe phenotype than the one outlined in this study, resulting in polycystic kidney disease and death by 28 days of age.^54^ The decrease of laminin α5 protein in this model appears to be greater than in our model and may explain the more severe phenotype.

There are several possible mechanisms whereby the altered GBM composition could cause the nephrotic phenotype. First, based on the electrokinetic filtration model, the electric field generated across the glomerular filtration barrier influences the passage of albumin through the glomerular filtration barrier, dragging it toward the capillary lumen by electrophoresis.^55^^,^^56^ The altered composition of the GBM in *Lama5*^E884G/E884G^ mice may cause a more fluid extracellular matrix, and interfere with the generation of a homogeneous streaming potential, resulting in reduced electrophoresis and therefore proteinuria. Second, podocytes are subjected to high circumferential wall stress and shear stress.^57^ Differences in GBM composition could weaken the GBM and subject the podocytes to increased mechanical stress. Third, given the reduced secretion of the laminin-521 heterotrimer, the endoplasmic reticulum stress could result in the gradual deterioration of the podocytes. However, we observed a reduced laminin-521 expression and proteinuria before morphologic changes in the podocytes, which suggests there is a gradual change in the fundamental properties of the GBM rather than disease phenotypes being secondary to podocyte dysfunction and death. Injection of human laminin-521 in *Lamb2*^–/–^ mice delayed the onset of the decline in renal function, but the accumulation of the injected laminin occurred only on the endothelial side of the GBM, leading the authors to suggest that this is not due to the role of laminin-521 in signaling to podocytes.^58^

In summary, recent identification of mutations in *LAMA5* in pediatric patients affected by NS, as well as the description of a syndromic developmental disorder,^59^ indicated that this gene is important in human health and is supported by our data. *LAMA5* should therefore be screened as a candidate in case of nephrotic patients with no other diagnosis. Of the 3 families under study, 2 carried mutations in the laminin α5 short arm, 1 of which resulted in the change of a glutamic acid into a glycine in the L4a domain. Although it is not the same position, the mutation we describe herein will serve as a useful tool to dissect disease mechanisms and test new treatments to alleviate symptoms. The modification of the phenotype by genetic background suggests a pathway that is influencing the response(s) to a defective GBM and may be important in a range of renal diseases.

## Disclosure

FWKT reports grants from Imperial College London, during the conduct of the study; consultancy fees and advisory board: Rigel Pharmaceuticals and Novartis; research grant support, including clinical trials: Boehringer Ingelheim, MedImmune, and Rigel Pharmaceuticals, outside the submitted work. RL reports grants from Wellcome Trust, during the conduct of the study; and personal fees from Retrophin, outside the submitted work. All the other authors declared no competing interests.
