# Supplementary material for: A novel model of nephrotic syndrome results from a point mutation in Lama5 and is modified by genetic background
Source: Kidney Int. 2022 Mar;101(3):527–40. doi: 10.1016/j.kint.2021.10.031 (PMC8883398; doi:10.1016/j.kint.2021.10.031)
Supplement: Supplementary File (PDF) [file mmc1.pdf]

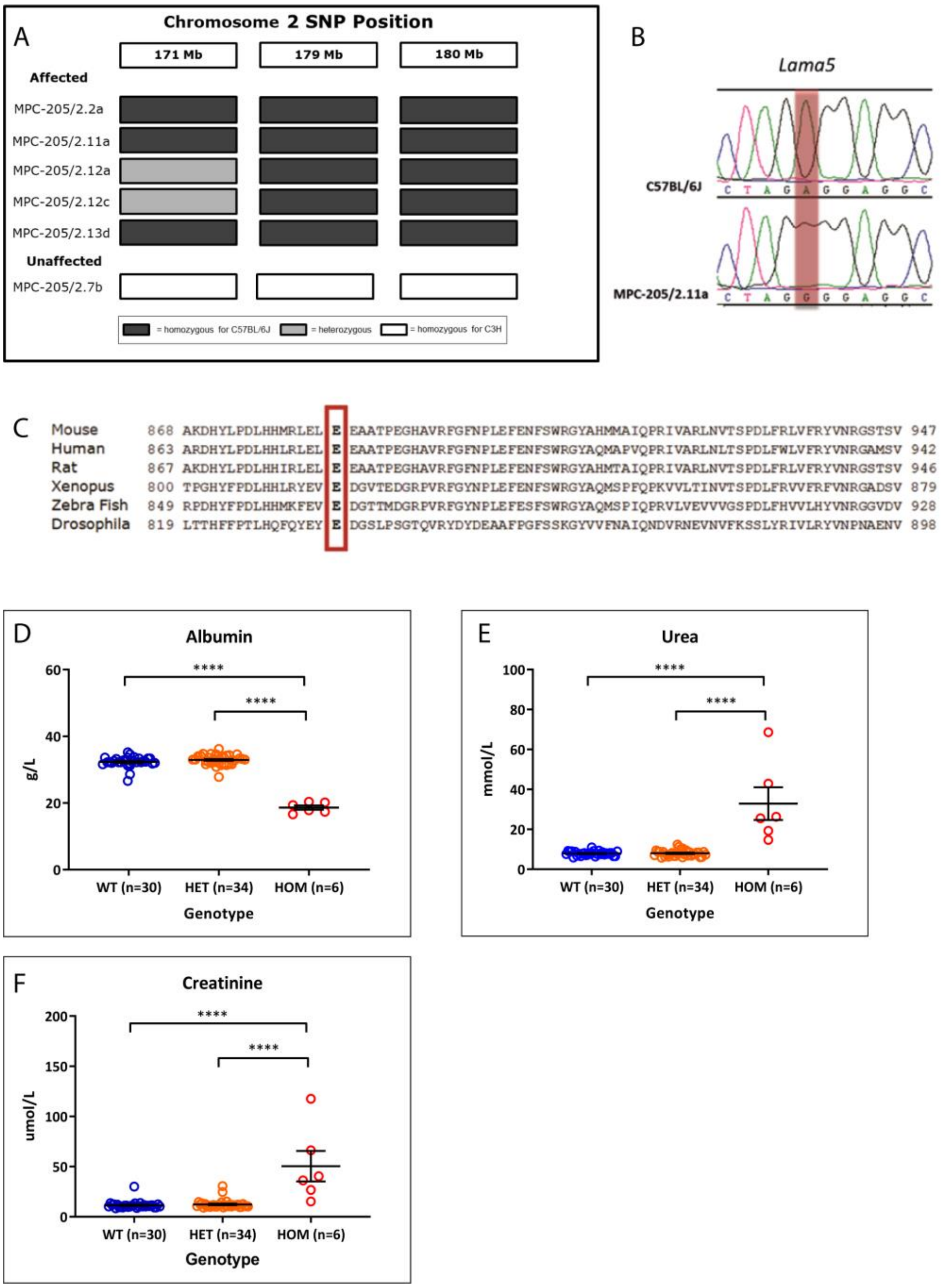

Supplementary Figure 1. **A**: Graphical representation of the candidate region on chromosome 2. **B**: Result of the Sanger sequencing of C57BL/6J control and an affected mouse from the MPC-205 line used to validate the mutation in *Lama5*. **C**: Comparison between the amino acid sequences of different species shows how the E884 residue is conserved down to Drosophila. **D-F:** Results of the clinical chemistry analysis of the pedigree MPC-205 at 6 months of age, with the animals grouped according to their genotype. The homozygous mice are the only ones showing alteration in the concentration of kidney markers (Albumin-**D**, Urea-**E** and Creatinine-**F**). The values shown are means ± SEM. One-way ANOVA **** p<0.0001.

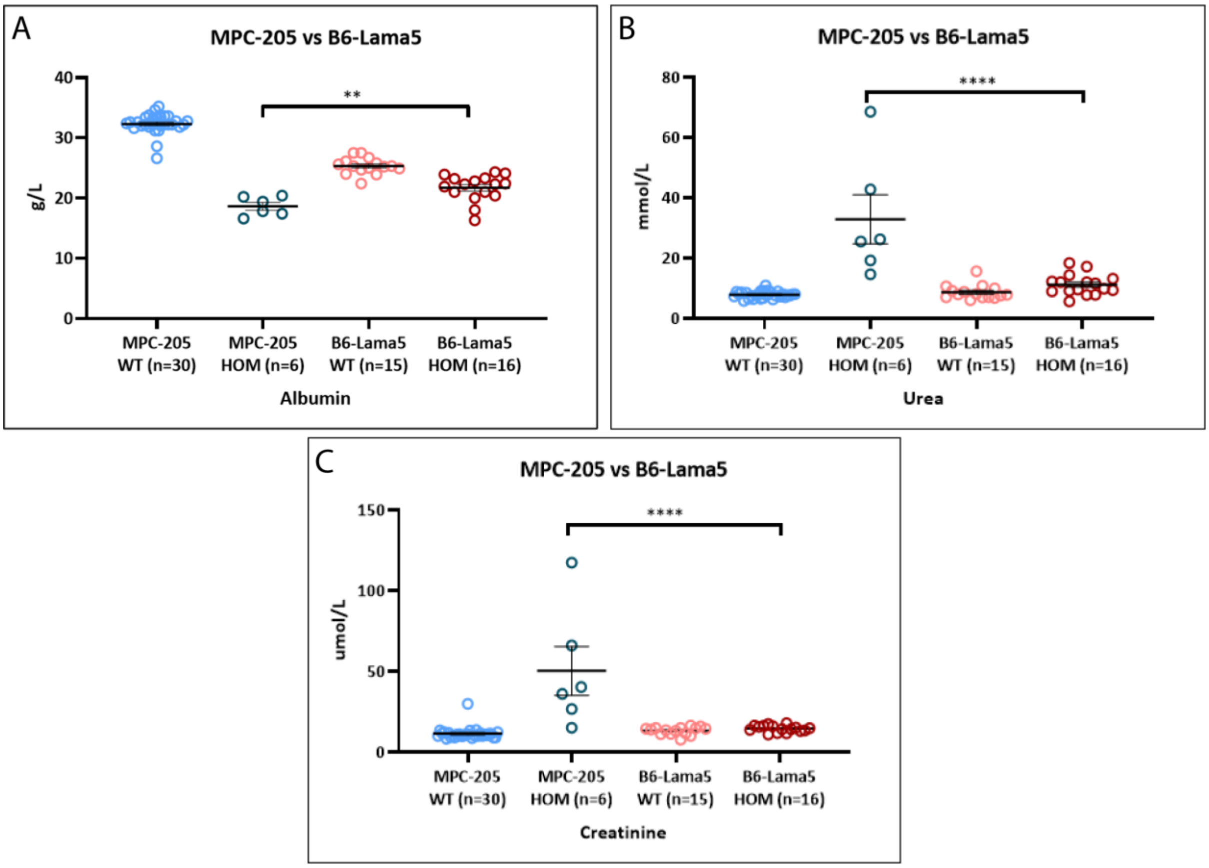

Supplementary Figure 2. Comparison of plasmatic albumin (**A**), urea (**B**) and creatinine (**C**) between the homozygous mice from the original pedigree and B6-*Lama5*$^{E884G/E884G}$ of the same age with respective wild type littermate controls. C57BL/6J congenic homozygous mice showed better kidney function when compared with 6 months old aged matched mixed background homozygotes (62.5% C57Bl/6J and 37.5% C3H.pde6).

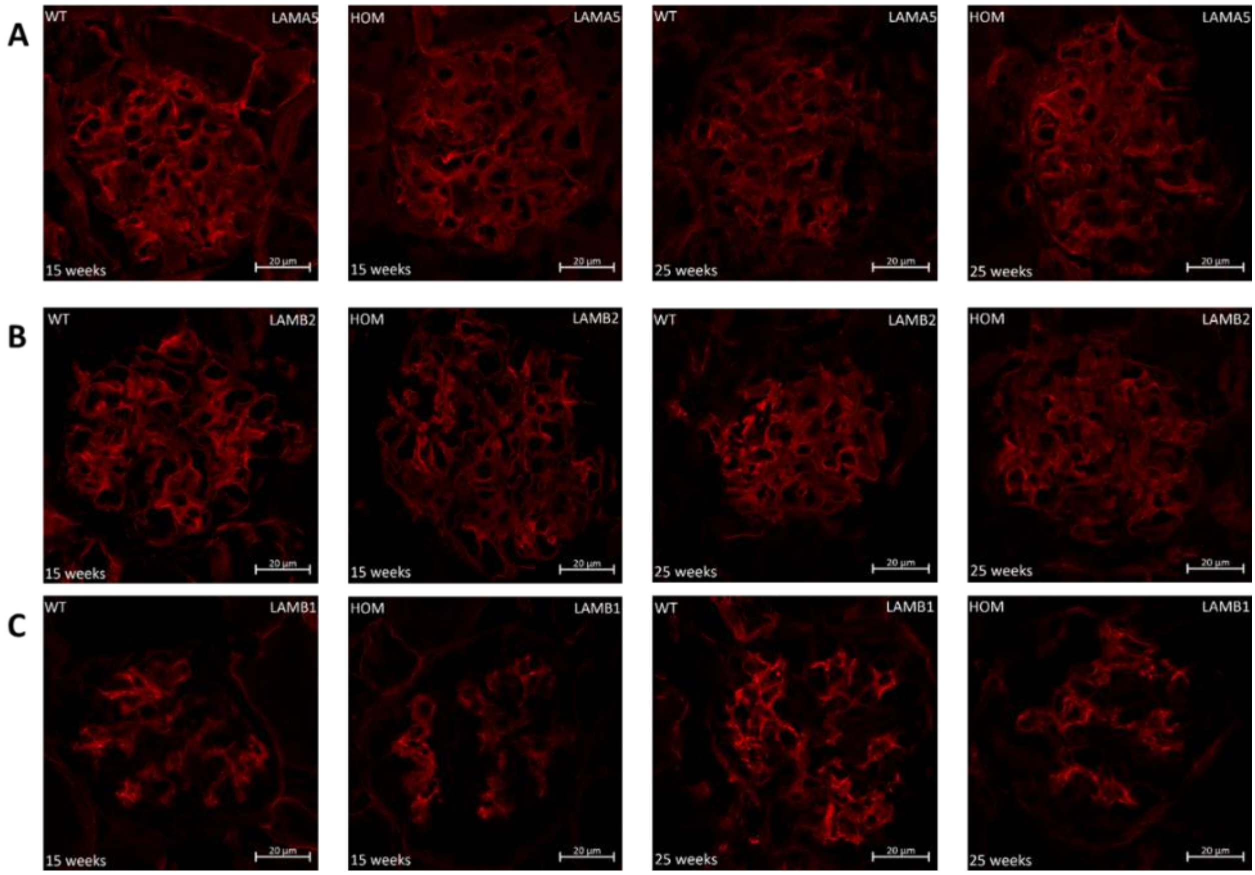

Supplementary figure 3. Representative immunofluorescence images of wild-type and homozygous mice, at 15 and 25 weeks, stained with anti-LAMA5 (**A**). anti-LAMB2 (**B**) and anti-LAMB1 (**C**). The mutant LAMA5 protein and its trimer partner LAMB2 are normally distributed in the homozygous sample suggesting a normal secretion and incorporation into the GBM. LAMB1 is expressed solely in the mesangium, demonstrating the absence of aberrant localization.

Chromosome	Position	Reference	Alteration	Functional Class	Gene Description	Gene Name
chr2	174640611	A	G	upstream_gene_variant	zinc finger protein 831	Zfp831
chr2	180630538	T	C	intron_variant	transcription factor-like 5 (basic helix-loop-helix)	Tcfl5
chr2	178110353	C	T	intergenic_variant	.	.
chr2	180827429	C	T	upstream_gene_variant	predicted gene 14340	.
chr2	178110363	T	C	intergenic_variant	.	.
chr2	179906554	T	A	intergenic_variant	.	.
chr2	181242002	T	C	5_prime_UTR_variant	cDNA sequence BC006779	BC006779
chr2	179379742	T	A	intergenic_variant	.	.
chr2	173488306	T	C	intergenic_variant	.	.
chr2	179187050	A	G	intergenic_variant	.	.
chr2	174153756	T	A	intron_variant	predicted gene 10714	.
chr2	178641261	T	C	intergenic_variant	.	.
chr2	180023388	T	C	upstream_gene_variant	LSM14 homolog B (SCD6, S. cerevisiae)	Lsm14b
chr2	173944795	C	T	intergenic_variant	.	.
chr2	179906558	T	A	intergenic_variant	.	.
chr2	181246554	C	T	upstream_gene_variant	cDNA sequence BC006779	BC006779
chr2	173944800	C	T	intergenic_variant	.	.
chr2	179252099	T	C	intergenic_variant	.	.
chr2	179381932	T	A	intergenic_variant	.	.
chr2	178818573	T	A	intergenic_variant	.	.
chr2	180196271	A	G	missense_variant	laminin, alpha 5	Lama5
chr2	174505629	T	C	intergenic_variant	.	.
chr2	178797493	G	T	intergenic_variant	.	.

Supplementary Table 1. List of the mutations included in the candidate region on chromosome 2. In light grey are shown the mutations with a low confidence score, in dark grey the mutations with a medium confidence score, in yellow the mutation with a high confidence score. *Lama5*^{E884G} missense variant is the only coding high confidence mutation resulting from the WGS analysis.

Variant	PROVEAN	SIFT	SNAP	Meta-SNP	PhD-SNP
E884G	-6.613<br>Deleterious<br>cutoff= < -2.5	0.000<br>Deleterious<br>cutoff= <0.5	0.725<br>Deleterious<br>cutoff= >0.5	0.691<br>Deleterious<br>cutoff= >0.5	0.587<br>Deleterious<br>cutoff= >0.5

Supplementary Table 2. Protein prediction software PROVEAN, SIFT, SNAP, Meta-SNP and PhD-SNP described the Lama5E884G mutation as deleterious.

		Protein:Creatinine Ration mg/mg creatinine	Urea (plasma) mmol/L	Creatinine (plasma) umol/L	Albumin (plasma) g/L	Tot Cholesterol (plasma) mmol/L	HDL (plasma) mmol/L	LDL (plasma) mmol/L
C3pde-*Lama5*	WT - 15 weeks	0.79 (0.10)	6.78 (0.55)	11.22 (0.73)	26.52 (0.46)	3.52 (0.17)	2.44 (0.11)	0.69 (0.07)
	HOM - 15 weeks	15.06 (2.53)	17.86 (2.96)	21.86 (4.02)	19.76 (0.67)	7.55 (0.36)	4.74 (0.32)	2.06 (0.15)
B6-*Lama5*	WT - 15 weeks	1.85 (0.13)	9.32 (0.59)	11.89 (0.46)	25.96 (0.43)	1.98 (0.12)	1.34 (0.10)	0.42 (0.02)
	HOM - 15 weeks	2.99 (0.48)	10.15 (0.75)	11.91 (0.64)	24.23 (0.38)	2.08 (014)	1.38 (0.10)	0.45 (0.03)
	WT - 25 weeks	2.08 (0.13)	8.75 (0.62)	13.33 (0.61)	25.33 (0.35)	2.08 (0.11)	1.42 (0.08)	0.52 (0.05)
	HOM - 25 weeks	26.41 (3.99)	11.24 (0.85)	14.68 (0.52)	21.70 (0.55)	3.02 (0.26)	2.01 (0.16)	0.75 (0.09)

Supplementary Table 3. Summary of urinary protein:creatinine ratio and clinical chemistry analysis on plasma of congenic 15 weeks old C3pde-*Lama5*, congenic 15 weeks old B6-*Lama5* and congenic 25 weeks old B6-*Lama5*. Values displayed represent average and SEM in brackets.
